# Editorial: Turning cold tumors hot: insights into molecular mechanisms and clinical applications of immunogenic cell death

**DOI:** 10.3389/fcell.2024.1496001

**Published:** 2024-09-24

**Authors:** Alessio Biagioni, Giulia Petroni, Cyril Corbet, Elena Andreucci

**Affiliations:** ^1^ Department of Experimental and Clinical Biomedical Sciences “Mario Serio”, University of Florence, Florence, Italy; ^2^ University of Florence, Department of Experimental and Clinical Medicine, Florence, Italy; ^3^ Pole of Pharmacology and Therapeutics (FATH), Institut de Recherche Expérimentale et Clinique (IREC), UCLouvain, Brussels, Belgium

**Keywords:** immunogenic cell death (ICD), damage-associated molecular patterns (DAMPs), tumor immunophenotype, tumor microenvironment (TME), anti-tumor immune responses, tumor neoantigens, cancer immunogenicity

Despite significant advancements in cancer therapy, treating cancer patients remains challenging mainly due to several therapy evasion mechanisms, which are still not well characterized, and severe side effects that limit the usage and clinical benefit of anti-cancer therapies. Since the implementation of immunotherapy, cancer research has been focused on understanding the complex heterogeneity of the tumor microenvironment (TME) to turn non-responsive—or “cold” tumors—into more treatable and responsive diseases—then defined as “hot”. In this context, accumulating data from preclinical and clinical studies have shown that numerous FDA-approved anticancer treatments can potentiate tumor immunogenicity and trigger anti-tumor immune responses (thereby boosting therapy effectiveness) through a plethora of mechanisms, including the so-called “immunogenic cell death” (ICD). As widely reviewed by Han et al., “cold” tumors are characterized by their low immunogenicity and an immunosuppressive TME, making them less responsive to conventional immunotherapies. In contrast, “hot” tumors have abundant immune cell infiltration and respond better to immunotherapy. The review addresses various traditional therapies, such as chemotherapy, targeted therapy, radiotherapy, and photodynamic therapy, demonstrated to induce ICD; it also highlights newly discovered and technologically innovative ICD inducers able to activate the immune system at the molecular level. Furthermore, the authors discuss the biological mechanisms and hallmarks of ICD, emphasizing its role in enhancing the immunogenicity of tumor cells. The authors also explore the clinical applications of combining ICD inducers with cancer immunotherapy, suggesting that this combination could improve the prognosis for patients with immune “cold” tumors. Moreover, Lu et al., and colleagues proposed a modern classification of accidental and regulated cell death forms, including necroptosis, ferroptosis, and pyroptosis, as typical ICDs. They brilliantly described the release of damage-associated molecular patterns (DAMPs) and tumor-associated antigens (TAAs) as key mediators of activation for dendritic cells and subsequent T cell priming, also defining the cascade of events occurring in the TME following necroptosis, ferroptosis, and pyroptosis. They lastly dissected different therapeutic approaches exploiting ICD in cancer cells by combining immune check-point blockade therapy with chemotherapy or photothermal and photodynamic therapy, or the release of reactive oxygen species triggered by radiation therapy, or by the use of novel immune-activating peptides. All these novel approaches might pave the way to developing new technologies for the discrimination of different regulated cell death programs that are commonly characterized by confluent pathways. Through this, these approaches could aid patient categorization for improved clinical therapies. Building on the exploration of ICD mechanisms and their therapeutic potential, Wen et al. focused on acute myeloid leukemia, which is a highly heterogeneous disease characterized by an uncontrolled proliferation of leukemic blasts, dividing two different cohorts of patients’ data collected from TGCA and GEO websites into two separated clusters based on 33 ICD genes expression levels. In samples grouped as high-ICD (cluster C2), the high expression of ICD-related genes—such as *NLRPJ*, *ENTPD1*, *TLR4*, *FNGR1*, *LY96*, *MYD8S*, *CASP1*, *P2RX7*, *CD4*, and *IL17RA*—was associated with a more immunoreactive phenotype enriched in gene signatures specific for B cells, CD8^+^ T cells, dendritic cells, macrophages, neutrophils, NK cells, CD4^+^ T cells, and Treg cells, suggesting that such a cluster might benefit from immunotherapy. Thanks to these observations, they identified 153 pseudogenes associated with the previous 33 ICD-related genes and, among them, 23 pseudogenes were found to be closely related to AML. By using 15 selected pseudogenes, they lastly generated a prognostic model exploitable in determining the clinical efficacy of immunotherapy and chemotherapy for AML patients. Another interesting approach is the one exploited by Wang et al., who studied the effects of different ablation temperatures on inducing ICD in hepatocellular carcinoma (HCC) cells. Ablation therapy, a common treatment for HCC, involves destroying cancer cells by applying extreme temperatures. The researchers assessed cell viability and apoptosis of four different HCC cell lines after *in vitro* exposure to ablation temperatures of −80°C, −40°C, 0°C, and 60°C, with 37°C serving as control. Additionally, they analyzed the expression of ICD-related cytokines, including calreticulin, ATP, high mobility group box 1 (HMGB1), and CXCL10. The authors demonstrated that apoptosis rates significantly increased in the −80°C and 60°C groups across all cell lines whilst the expression levels of ICD-related cytokines varied significantly between the different temperature groups. In particular, calreticulin expression was higher in the 60°C group and lower in the −80°C group for Hepa1-6 and SMMC7221 cells, while ATP, HMGB1, and CXCL10 levels were elevated in all cell lines after exposure to 60°C, −80°C, and −40°C. The study concludes that different ablation temperatures can induce distinct types of ICDs in HCC cells, suggesting a potential strategy for personalized cancer therapies. By tailoring ablation temperatures, it may be possible to optimize ICD induction and improve the effectiveness of cancer treatments.

